# On Shape Optimization with Large Magnetic Fields in Two Dimensions

**DOI:** 10.1007/s12220-026-02363-7

**Published:** 2026-03-07

**Authors:** Vladimir Lotoreichik, Léo Morin

**Affiliations:** 1 Department of Theoretical Physics, Nuclear Physics Institute, Czech Academy of Sciences, 25068 Řež, Czech Republic; 2 Department of Mathematics, University of Copenhagen, Universitetsparken 5, DK-2100 Copenhagen Ø, Denmark

**Keywords:** Spectral theory, Shape optimization, Magnetic Laplacian, 35P15, 35Q40, 58J50

## Abstract

This paper aims to show that, in the limit of strong magnetic fields, the optimal domains for eigenvalues of magnetic Laplacians tend to exhibit symmetry. We establish several asymptotic bounds on magnetic eigenvalues to support this conclusion. Our main result implies that if, for a bounded simply-connected planar domain, the *n*-th eigenvalue of the magnetic Dirichlet Laplacian with uniform magnetic field is smaller than the corresponding eigenvalue for a disk of the same area, then the Fraenkel asymmetry of that domain tends to zero in the strong magnetic field limit. Comparable results are also derived for the magnetic Dirichlet Laplacian on rectangles, as well as the magnetic Dirac operator with infinite mass boundary conditions on smooth domains. As part of our analysis, we additionally provide a new estimate for the torsion function on rectangles.

## Introduction

### Background and Motivation

The optimization of eigenvalues of differential operators with respect to the shape of a domain is a central theme in spectral geometry, featuring numerous intriguing results alongside challenging open questions. For the usual Laplacian with Dirichlet boundary conditions, the Faber-Krahn inequality [22, 34, 35] states that, among all bounded domains of fixed volume, the ball minimizes the lowest eigenvalue. In two dimensions, L. Erdős [21] extended this inequality to the magnetic Laplacian with homogeneous magnetic field. A recent work [26] further strengthened Erdős’s result by providing a quantitative version. However, the problem of eigenvalue optimization for the magnetic Laplacian under other boundary conditions and for higher eigenvalues remains far from fully understood. Partial progress has been made in maximizing the lowest eigenvalue of the magnetic Laplacian with Neumann [18, 32] and Robin [31] boundary conditions. In particular, the article [18] settles the optimization problem for the lowest eigenvalue under Neumann boundary conditions in the regime of moderate magnetic fields.

It was recently conjectured by Baur [10]–based on numerical evidence–that for any eigenvalue of the magnetic Dirichlet Laplacian on bounded planar domains of fixed area, the disk is the optimal shape whenever the magnetic flux exceeds an explicit critical threshold. This observation is in shear contrast with the non-magnetic case, where the disk minimizes only the lowest eigenvalue, and is conjectured to optimize the third eigenvalue [29, Open problem 8]. For other eigenvalues of the usual Dirichlet Laplacian on planar domains, the disk is never an optimizer, a fact which is verified analytically in [11] by computing shape derivatives (see also the numerics from [5, 42]).

Our aim in this note is to obtain asymptotic results supporting the conjecture of Baur. Moreover, we observe a broader pattern: optimal domains for magnetic eigenvalues tend to become symmetric as the magnetic field strength grows. We further illustrate this principle in the contexts of the magnetic Laplacian on rectangles and magnetic Dirac operators with infinite mass boundary conditions.

### Statement of the Results

Let $\Omega \subset \mathbb {R}^2$ be a bounded simply-connected planar domain. At this stage no regularity of the boundary of $\Omega$ is assumed. We consider the shifted magnetic Laplacian with Dirichlet boundary conditions^1^

$$ \mathscr {L}_{\Omega ,B}u := -(\nabla -i \textbf{A})^2u -Bu, \qquad \textrm{dom}\,\mathscr {L}_{\Omega ,B} := \big \{u\in H^1_0(\Omega ):\Delta u \in L^2(\Omega )\big \}. $$

Here the vector field $\textbf{A}= (A_1,A_2)^\top$ generates a homogeneous magnetic field of strength $B\ge 0$, i.e. $\partial _1 A_2 - \partial _2 A_1 = B$ and for the sake of definiteness one can use the Landau gauge $\textbf{A}= (-Bx_2,0)^\top$ in the definition of the operator $\mathscr {L}_{\Omega ,B}$. Since the domain $\Omega$ is simply-connected, the spectrum of $\mathscr {L}_{\Omega ,B}$ does not depend on the gauge of the vector potential, at least for regular enough vector potential and boundary of $\Omega$. In the proofs, it is more convenient to use the gauge associated with the torsion function rather than the Landau gauge. The torsion function–based gauge will be introduced later. For a comprehensive treatment of the spectral theory of the magnetic Laplacian, the reader is referred to the monographs [24, 45].

The operator $\mathscr {L}_{\Omega ,B}$ is self-adjoint with compact resolvent, and it has a non-decreasing sequence of eigenvalues,

$$ 0<\lambda _1(\Omega ,B) \le \lambda _2(\Omega ,B) \le \cdots , $$

which are repeated with multiplicities. It is well known that the first eigenvalue is positive: it follows from [28, Theorem 3.1] for instance, see also Lemma 8 below. We are interested in the dependence on $\Omega$ of these eigenvalues when *B* is large. Our work is in fact motivated by the following conjecture from the recent article [10], which is supported by numerics.

#### Conjecture 1

([10, Conjecture 4.2]) Let $\Omega \subset \mathbb {R}^2$ be a bounded simply-connected planar domain with area $|\Omega |=1$, and $n \ge 2$. Then, for all $B\ge 2\pi n$,

1.1 $$\begin{aligned} \lambda _n(\Omega ,B) \ge \lambda _n(\mathbb {D},B), \end{aligned}$$

where $\mathbb {D}$ is the disk of unit area.

We remark that the assumption of unit area can be made without loss of generality, since the general case follows by scaling. For domains of general area, the condition on *B* should be replaced by the respective condition on the flux, $B |\Omega | \ge 2\pi n$. As explained in [10], the above conjecture illustrates the strong influence of magnetic fields on shape optimization problems. Indeed, when $B=0$, the minimizing shape of the Laplace eigenvalue $\lambda _n(\Omega ,0)$ is typically not a disk except for $n=1$ and conjecturally for $n=3$. In fact, the numerics from [10] suggest that the lower bound $B \ge 2\pi n$ is sharp. Conjecture 1 is also supported by the fact that explicit computations proposed in [10, Appendix A] can be used to show that, when *B* exceeds $2\pi n$ the single disk starts to be the minimizer of the *n*-th magnetic Dirichlet eigenvalue among all disjoint unions of disks of different radii with fixed total area.

As an attempt of getting more insights on this conjecture, we explain in the present article how to get asymptotic results when *B* is large. More precisely, we show that any minimizing shape of $\lambda _n(\Omega ,B)$ must converge to a disk as $B \rightarrow \infty$. This is measured in terms of the Fraenkel asymmetry,

1.2 $$\begin{aligned} \alpha (\Omega ) = \inf _{x \in \mathbb {R}^2} \frac{|\Omega \Delta (\mathcal {D}+ x)|}{|\Omega |}, \end{aligned}$$

where $\mathcal {D}$ is the disk of the same area as $\Omega$, and $\Delta$ is the symmetric difference of sets *i.e.* $A\Delta B:= (A\setminus B)\cup (B\setminus A)$ for $A,B\subset {\mathbb R}^2$. The Fraenkel asymmetry is widely used in quantitative spectral isoperimetric inequalities; see the review [9] and the references therein. We prove the following lower bound on the magnetic eigenvalues.

#### Theorem 1

For all $n \in \mathbb {N}$, there exist $c_n,B_n >0$ such that the following holds. If $\Omega \subset \mathbb {R}^2$ is a bounded simply-connected planar domain of unit area, then for all $B \ge B_n$,

$$\begin{aligned} \frac{\lambda _n(\Omega ,B)}{\lambda _n(\mathbb {D},B)} \ge 1 + c_n B \alpha (\Omega )^3 - \frac{\ln B}{c_n}, \end{aligned}$$

where $\mathbb {D}$ is the disk of unit area.

We emphasize that this theorem does not imply Conjecture 1, even for large values of *B*. However, it supports the conjecture, by showing that optimal domains should asymptotically converge to a disk. Indeed, if $\Omega _B\subset {\mathbb R}^2$ is a unit area bounded simply-connected domain such that $\lambda _n(\Omega _B,B) \le \lambda _n(\mathbb {D},B)$ then it should have small asymmetry,

$$\begin{aligned} \alpha (\Omega _B)^3 \le \frac{\ln B}{c_n^2 B}, \end{aligned}$$

for all *B* sufficiently large.

In fact, our arguments also work for other shape optimization problems with strong magnetic field. For instance, we have a similar result on the optimization of $\lambda _n(\Omega ,B)$ among rectangles, thus showing that an optimizer asymptotically converges to a square in the strong magnetic field limit.

#### Theorem 2

For all $n \in \mathbb {N}$, there exist $c_n,B_n>0$ such that the following holds. If $a>0$ and $\textsf {R}_a$ is the rectangle of side lengths *a* and $a^{-1}$, then for all $B \ge B_n$,

$$\begin{aligned} \frac{\lambda _n(\textsf {R}_a,B)}{\lambda _n(\textsf {R}_1,B)} \ge 1 + c_n B \frac{(a^2-1)^2}{1+a^4} - \frac{\ln B}{c_n}. \end{aligned}$$

The question of optimality of the square for the first magnetic eigenvalue was recently raised in [37]. Theorem 2 supports the conjecture from [37] that the square should always be optimal. However, Theorem 2 only gives asymptotic convergence of the optimal rectangle to a square. Indeed, if $\textsf {R}_a$ is such that $\lambda _n(\textsf {R}_a,B) \le \lambda _n(\textsf {R}_1,B)$ then we deduce that

$$\begin{aligned} \frac{(a^2-1)^2}{1+a^4} \le \frac{\ln B}{c_n^2 B}, \end{aligned}$$

for $$B \ge B_n$$.

We remark that asymptotic optimality of the square was also shown for the principal eigenvalue of the Dirac operator on a rectangle with infinite mass boundary conditions in the large mass limit [14]. The spectral optimization for rectangles is also studied for the biharmonic operator [15], where variables cannot be separated.

Finally, we also get similar estimates for magnetic Dirac operators, thus suggesting that Conjecture 1 could have an analogue for such operators. The non-magnetic Dirac operator with infinite mass boundary conditions on $\Omega$ was introduced in [12], where self-adjointness of this operator was established. The introduction of this operator was partly motivated by the study of graphene quantum dots. Several later works [16, 17, 38, 43] were concerned with defining this operator on certain classes of non-smooth domains allowing for corners. In the non-magnetic setting, geometric bounds and optimization of the smallest positive eigenvalue of the Dirac operator with infinite mass boundary conditions on Euclidean domains were studied in [3, 4, 13, 14, 20, 41]. A counterpart of the Faber-Krahn inequality in the case of Dirac operators on Euclidean domains remains an open problem, but there is a numerical evidence in [3] for the validity of this inequality, see also more recent numerical study for higher eigenvalues in [4].

In the magnetic case, a semi-classical analysis of the operator was performed in [8, 39]. We adopt some notation used therein. In order to introduce the Dirac operator, we recall the definition of Cauchy-Riemann operators

1.3 $$\begin{aligned} \partial _{\overline{z}} = \frac{\partial _1+i\partial _2}{2}\qquad \text {and}\qquad \partial _z = \frac{\partial _1-i\partial _2}{2} \end{aligned}$$

and define the first-order operators

1.4 $$\begin{aligned} d_\textbf{A} := -2i\partial _z - A_1+i A_2,\qquad d_\textbf{A}^\times := -2i\partial _{\overline{z}} - A_1-i A_2, \end{aligned}$$

where as before $A_1$ and $A_2$ are the components of the magnetic vector potential $\textbf{A}$. Let $\Omega \subset {\mathbb R}^2$ be a bounded, simply-connected $\mathscr {C}^2$-smooth domain. We denote by $\nu = (\nu _1,\nu _2)$ the outer unit normal vector to the boundary of $\Omega$. We remark that $\mathscr {C}^2$-smoothness of the domain simplifies the definition of the Dirac operator and allows to use methods, which are only available for more regular domains. In view of [12, Theorem 1.1] the magnetic Dirac operator

1.5 $$\begin{aligned} \begin{aligned} \mathscr {D}_{\Omega ,B} u&:= \begin{pmatrix} 0 &  d_\textbf{A} \\ d_\textbf{A}^\times &  0 \end{pmatrix} u,\\ \textrm{dom}\,\mathscr {D}_{\Omega ,B}&:= \big \{u = (u_1,u_2)\in H^1(\Omega ;{\mathbb C}^2):u_2 = i(\nu _1+i\nu _2) u_1~ \mathrm{{on}}~\partial \Omega \big \} \end{aligned} \end{aligned}$$

is self-adjoint on the Hilbert space $L^2(\Omega ;{\mathbb C}^2)$. Here, we implicitly used that adding the magnetic field leads to a bounded symmetric additive perturbation of the non-magnetic Dirac operator with infinite mass boundary conditions. Thus, adding the magnetic field does not influence self-adjointness. The spectrum of this Dirac operator is purely discrete thanks to compact embedding of $H^1(\Omega )$ into $L^2(\Omega )$. By [8, Proposition 1.5] we have $0\notin \sigma (\mathscr {D}_{\Omega ,B})$. It is clear that the magnetic field manifests in the spectrum of $\mathscr {D}_{\Omega ,B}$ and we denote by $\lambda _n^+(\Omega ,B)$ (respectively by $\lambda _n^-(\Omega ,B)$) the positive (respectively, negative) eigenvalues of $\mathscr {D}_{\Omega ,B}$ enumerated in non-decreasing (respectively, non-increasing) way and counted with multiplicities,

$$ \dots \le \lambda _2^-(\Omega ,B)\le \lambda _1^-(\Omega ,B)< 0 < \lambda _1^+(\Omega ,B)\le \lambda _2^+(\Omega ,B)\le \dots $$

We prove the following bound on the positive eigenvalues of $\mathscr {D}_{\Omega ,B}$.

#### Theorem 3

For all $n \in \mathbb {N}$, there exist $c_n,B_n >0$ such that the following holds. If $\Omega \subset \mathbb {R}^2$ is a bounded, simply-connected, $\mathscr {C}^2$-smooth domain with unit area, then for all $B \ge B_n$,

$$\begin{aligned} \frac{\lambda _n^+(\Omega ,B)}{\lambda _n^+(\mathbb {D},B)} \ge 1 + c_n B \alpha (\Omega )^3 - \frac{\ln B}{c_n}. \end{aligned}$$

We remark that the spectrum of the Dirac operator $\mathscr {D}_{\Omega ,B}$ is not symmetric with respect to the origin for non-zero magnetic field. Moreover, in the large magnetic field limit, the eigenfunctions corresponding to its positive eigenvalues are localized near the maximum of the torsion function for $\Omega$, while the eigenfunctions corresponding to its negative eigenvalues are localized near the boundary [8, Remark 1.18]. Due to such a substantial difference, we do not expect a direct counterpart of Theorem 3 for the negative eigenvalues of $\mathscr {D}_{\Omega ,B}$.

The proofs of Theorems 1, 2 and 3 follow the same strategy, and rely on three ingredients:

- Upper bounds on the *n*-th eigenvalue of the optimizing shape (i.e. on $\lambda _n(\mathbb {D},B)$, $\lambda _n(\textsf {R}_1,B)$ and $\lambda _n^+(\mathbb {D},B)$) that are asymptotically sharp when *B* is large. These bounds are given in Lemmata 7 and 9, respectively, and come from [7, 8]. The bound in Lemma 7 is stated for general convex domains and applies to both ${\mathbb D}$ and ${\textsf {R}}_1$.
- Lower bounds on $\lambda _1(\Omega ,B)$ and $\lambda _1^+(\Omega ,B)$ that are uniform with respect to $\Omega$, and still catch the exponentially small behaviour as *B* increases. For the magnetic Laplacian, this bound comes from [28] (see Lemma 8). In the case of the magnetic Dirac operator, we adapt the argument to prove Lemma 10.
- In these asymptotic estimates, the torsion function $\varphi ^\Omega$ (defined in Section 2) plays a crucial role. In fact, the smallness of the eigenvalues is measured in terms of the maximum of the torsion function. It follows from Talenti’s comparison principle that $\Vert \varphi ^\Omega \Vert _\infty$ is maximized by the disk among domains of fixed area. We need a quantitative version of this result, see Lemma 4, which can also be derived as a consequence from more general recent quantitative Talenti’s comparison principle [1, 2]. In the case of rectangles, we prove that the square maximizes $\Vert \varphi ^\Omega \Vert _\infty$ in Theorem 5, also in quantitative form. The latter result on the torsion function is new to the best of our knowledge.

It remains to outline the structure of the paper. Section 2 is devoted to the torsion function and to quantitative isoperimetric inequalities for its maximal value. We prove Theorems 1 and 2 in Section 3 while the proof of Theorem 3 is given in Section 4. The paper is complemented by an appendix, where we provide a new proof of an essentially known result on the torsion function, which is central for our analysis.

## On the Maximum of the Torsion Function

The spectra of the magnetic Dirichlet Laplacian $\mathscr {L}_{\Omega ,B}$ and of the magnetic Dirac operator $\mathscr {D}_{\Omega ,B}$ in strong magnetic fields are deeply related to the *torsion function* $\varphi ^\Omega$. This function is defined as the solution to the elliptic equation

2.1 $$\begin{aligned} {\left\{ \begin{array}{ll} -\Delta \varphi ^\Omega = 1 & \quad \textrm{in} \quad \Omega ,\\ \varphi ^\Omega = 0 & \quad \textrm{on} \quad \partial \Omega . \end{array}\right. } \end{aligned}$$

Note that this elliptic equation has a unique solution in the Sobolev space $H^1_0(\Omega )$. By the maximum principle, the function $\varphi ^\Omega$ is positive inside $\Omega$, and we denote by $\varphi ^\Omega _\textrm{m}$ its maximum.

It is standard that the maximum of the torsion function is always maximized by the disk among domains of fixed area. This follows from Talenti’s comparison principle [48, Theorem 1]. In fact, we also have a quantitative version of this result, which takes into account the Fraenkel asymmetry $\alpha (\Omega )$ defined in (1.2).

### Lemma 4

There is a $c >0$ such that, for all bounded open sets $\Omega \subset \mathbb {R}^2$,

$$\begin{aligned} \varphi _\textrm{m}^{\mathcal {D}} - \varphi _\textrm{m}^\Omega \ge c |\Omega | \alpha (\Omega )^3 \end{aligned}$$

where $\mathcal {D}\subset {\mathbb R}^2$ is a disk of the same area as $\Omega$.

### Remark 1

Our analysis combined with a bound in [40] on the best constant in the quantitative isoperimetric inequality [25] used in the proof of the above lemma yields as a by-product that we can choose $c = \frac{1}{1024\pi }$.

The cubic-power term $\alpha (\Omega )^3$ on the right-hand side could probably be improved to $\alpha (\Omega )^2$, but we do not need such a precise result for our purposes. In fact, this improvement is considered to be an important open problem; see the discussion in [1]. Lemma 4 was first obtained in [33], using probabilistic methods. For completeness we suggest the proof outsourced to the appendix and inspired by [2, Theorem 1.2]. In fact, this result in [2] uses ideas from [27], which were also adapted to the magnetic setting in [26].

Lemma 4 has a counterpart for rectangular domains. The main additional ingredient in the case of rectangles is the fact that $\varphi ^{\textsf {R}_a}_\textrm{m}$ is maximized by the square ($a=1$). We prove the following stronger quantitative estimate on the maximum of the torsion function on rectangles $\textsf {R}_a=(-a/2,a/2)\times (- \frac{1}{2a},\frac{1}{2a})$. This property of the torsion function on rectangles is new to the best of our knowledge.

### Theorem 5

For all $a>0$ we have

$$\begin{aligned} \varphi _\textrm{m}^{\textsf {R}_1} - \varphi _\textrm{m}^{\textsf {R}_a} \ge \frac{(a^2-1)^2}{24(1+a^4)}. \end{aligned}$$

### Proof

The torsion function of the rectangle $\textsf {R}_a$ is explicitly given by

$$\begin{aligned} \varphi ^{\textsf {R}_a}(x_1,x_2) = \frac{16}{\pi ^4} \sum _{n, m \ge 0} \frac{\sin \Big ( \frac{(2n+1)\pi }{2a}(2x_1+a) \Big ) \sin \Big ( \frac{(2m+1)\pi a}{2}(2x_2+ \frac{1}{a}) \Big )}{(2n+1)^3(2m+1)} \frac{a^2}{1+ a^4\frac{(2m+1)^2}{(2n+1)^2}}, \end{aligned}$$

as one can find using Fourier series; *cf.* [6, Section 7]. Note that this series is uniformly absolutely convergent. Indeed, if we bound the sine functions by 1, we get a series

$$\begin{aligned}  &   \frac{16}{\pi ^4} \sum _{n, m \ge 0} \frac{1}{(2n+1)^3(2m+1)} \frac{a^2}{1+ a^4\frac{(2m+1)^2}{(2n+1)^2}} \\  &   \quad = \frac{16}{\pi ^4} \sum _{n, m \ge 0} \frac{1}{(2n+1)^2(2m+1)^2} f \Big ( a^2 \frac{2m+1}{2n+1} \Big ), \end{aligned}$$

where $f(x) = \frac{x}{1+x^2}$, and this series converges because $|f|\le \frac{1}{2}$.

We also recall that, for any convex domain, the square root of the torsion function is strictly concave [36, Theorem 1] and thus the torsion function has a unique global maximum. In the case of a rectangle, by symmetry, the maximum should be reached at the center $x=0$. Thus,

$$\begin{aligned} \varphi _\textrm{m}^{\textsf {R}_a} = \frac{16}{\pi ^4} \sum _{n,m \ge 0} \frac{(-1)^{n+m}}{(2n+1)^2(2m+1)^2}f \Big ( a^2 \frac{2m+1}{2n+1} \Big ). \end{aligned}$$

We extract from this sum the diagonal terms $m=n$,

$$\begin{aligned} \varphi _\textrm{m}^{\textsf {R}_a}= &   \frac{16}{\pi ^4} \sum _{n \ge 0} \frac{1}{(2n+1)^4} f(a^2) + \frac{16}{\pi ^4} \sum _{m >n} \frac{(-1)^{n+m}}{(2n+1)^2(2m+1)^2}\\  &   \times \left( f \left( a^2 \frac{2m+1}{2n+1} \right) + f \left( a^2 \frac{2n+1}{2m+1} \right) \right) . \end{aligned}$$

Therefore, we have to get a lower bound for

$$\begin{aligned} \varphi _{\textrm{m}}^{\textsf {R}_1} - \varphi _{\textrm{m}}^{\textsf {R}_a}= &   \frac{16}{\pi ^4} \sum _{n \ge 0} \frac{1}{(2n+1)^4} \big ( f(1)-f(a^2) \big ) \\  &   +\frac{16}{\pi ^4}\sum _{m >n} \frac{(-1)^{n+m}}{(2n+1)^2(2m+1)^2} g \left( a^2, \frac{2m+1}{2n+1} \right) , \end{aligned}$$

where $g(x,y) = 2f(y) - f(xy) - f(x/y)$. By Lemma 6 below, the rational function *g* is bounded by $|g(x,y)| \le g(x,1) = 2(f(1)-f(x))$. Thus,

$$\begin{aligned} \varphi _{\textrm{m}}^{\textsf {R}_1} - \varphi _{\textrm{m}}^{\textsf {R}_a} \ge \frac{16}{\pi ^4} \left( \sum _{n \ge 0} \frac{1}{(2n+1)^4} - \sum _{m \ne n} \frac{1}{(2n+1)^2(2m+1)^2} \right) \big ( f(1)-f(a^2) \big ). \end{aligned}$$

We recall that

$$\begin{aligned} \sum _{n\ge 0} \frac{1}{(2n+1)^4} = \frac{15}{16} \zeta (4) = \frac{\pi ^4}{96}, \quad \sum _{m \ne n} \frac{1}{(2n+1)^2(2m+1)^2} = \frac{\pi ^4}{192}. \end{aligned}$$

We deduce that

$$\begin{aligned} \varphi _{\textrm{m}}^{\textsf {R}_1} - \varphi _{\textrm{m}}^{\textsf {R}_a} \ge \frac{1}{12} (f(1)-f(a^2)) = \frac{(1-a^2)^2}{24(1+a^4)}. \end{aligned}$$

$$\square $$

To prove Theorem 5, we used a technical bound on a specific rational function.

### Lemma 6

Define the rational function *g* by

$$\begin{aligned} g(x,y) = \frac{2y}{1+y^2} - \frac{xy}{1+x^2y^2} - \frac{xy}{x^2+y^2}. \end{aligned}$$

Then, for all $x,y>0$,

$$\begin{aligned} |g(x,y)| \le g(x,1). \end{aligned}$$

### Proof

First, note that we can assume $x,y \le 1$ because of the symmetry $g(x,y) = g(x^{-1},y) = g(x,y^{-1})$. The function *g* can be factorized as

2.2 $$\begin{aligned} g(x,y) = \frac{y(1-x)^2(2x^2y^2 + 2y^2 - x(y^2-1)^2)}{(1+y^2)(1+x^2y^2)(x^2+y^2)}, \end{aligned}$$

and for $y=1$ we find

$$\begin{aligned} g(x,1) = \frac{(1-x)^2}{1+x^2}. \end{aligned}$$

In particular, we can factorize the difference as

$$\begin{aligned} g&(x,1)-g(x,y) = \\  &\frac{(1-x)^2(1-y)^2[x^4y^2 + x^3y(1+y)^2 + x^2 (1+2y+4y^2+2y^3+y^4) +xy(1+y)^2+y^2]}{(1+x^2)(1+y^2)(x^2+y^2)(1+x^2y^2)}, \end{aligned}$$

which is non-negative. Similarly, we find

$$\begin{aligned} g&(x,1)+g(x,y) =\\  &\frac{(1-x)^2(1+y)^2[x^2y^2(1-y)^2 + [x(1-y) - (1+x^2)y]^2 + x(1+x^2)y(1-y^2)]}{(1+x^2)(1+y^2)(x^2+y^2)(1+x^2y^2)}, \end{aligned}$$

which is obviously non-negative for $0 < y\le 1$. $\square$

## Eigenvalues of the Magnetic Laplacian

In the limit of large magnetic fields, it is known that the eigenfunctions of the magnetic Laplacian $\mathscr {L}_{\Omega ,B}$ are exponentially localized near the maximum points of the torsion function $\varphi ^\Omega$. In fact, this was one of the crucial observations in [7], where asymptotics of the form

$$\begin{aligned} C_n' B^{n+1} e^{-2B \varphi _\textrm{m}^\Omega } \le \lambda _n(\Omega ,B) \le C_n'' B^{n+1} e^{-2B \varphi _\textrm{m}^\Omega }, \quad \text {for }B\text { large enough,} \end{aligned}$$

were proven^2^. This result was the origin of our ideas to prove Theorem 1. However, the bounds in [7] are far from uniform with respect to $\Omega$, hence it is impossible to apply them directly in our setting. We shall only use the upper bound, in the specific cases of a disk or square, but we will state the bound for more general convex domains.

### Lemma 7

Let $n \in \mathbb {N}$ and let $\Omega \subset {\mathbb R}^2$ be a bounded convex domain. Then there exist $M_n = M_n(\Omega ) >0$ and $C_n = C_n(\Omega ) >0$ such that

$$\begin{aligned} \lambda _n(\Omega ,B) \le C_n B^{n+1} e^{-2B \varphi _\textrm{m}^\Omega }, \end{aligned}$$

for all $$B \ge M_n$$.

The proof of the above lemma is essentially given in [7] and even with a sharp asymptotic constant $C_n$ together with the corresponding lower bound. We present here a simpler version of the argument from [7], in which we do not aim at a sharp constant. Thanks to this simplification we could reduce the regularity assumptions on the boundary of the domain compared to [7]. We also point out that convexity of $\Omega$ is only used to guarantee that the torsion function of $\Omega$ has a unique global maximum with negative definite Hessian at the maximum point. We can instead assume that the domain $\Omega$ is such that the desired property of the torsion function holds, enlarging thus the class of domains to which the lemma applies. This extension is not needed for our purposes since we intend to apply this lemma only to disks and squares (both being convex domains). Note however that bounded convex domains always have a Lipschitz boundary, which is important in the proof.

### Proof of Lemma 7

Recall that, for any convex domain, the square root of the torsion function is strictly concave [36, Theorem 1] and thus the torsion function has a unique global maximum. Moreover, the Hessian of the torsion function at the point of the maximum is negatively definite [47, Theorem 1].

The proof is based on a variational principle for $\lambda _n(\Omega ,B)$ (equation (3.2)), together with the construction of quasimodes. The intuition behind this construction, which is already key in [7], is that the eigenfunctions should be exponentially localized near the maximum of the torsion function. This suggests to construct quasimodes of the form $\Psi _n(x) = e^{- B (\varphi ^\Omega _\textrm{m} - \varphi ^\Omega (x)) } p_n(x)$. Then, the variational formulation for $p_n$ (or $v_n = e^{- B \varphi ^\Omega _\textrm{m}}p_n$) suggests to choose $p_n$ holomorphic in $z=x_1+ix_2$: we take the simplest possible polynomials. However, in order to match the boundary condition, we need to add a cutoff function. In [7], this procedure is improved by optimizing the choice of polynomials and cutoff functions.

*Step 1: A variational formulation.* Without loss of generality we can assume that the domain $\Omega$ is positioned so that the unique maximum of its torsion function is attained at the origin. Note that since $\Omega$ is convex and, in particular, simply connected, we can choose the specific gauge of the magnetic field

3.1 $$\begin{aligned} A_1 = B \partial _2 \varphi ^\Omega , \qquad A_2 = - B \partial _1 \varphi ^\Omega , \end{aligned}$$

which indeed satisfies $\partial _1 A_2 - \partial _2 A_1 = B$. Then, with the notations (1.3) and (1.4) we formally have

$$\begin{aligned} \mathscr {L}_{\Omega , B} = -(\nabla - i\textbf{A})^2 - B = d_{\textbf{A}}  d^\times _{\textbf{A}}. \end{aligned}$$

In particular, the quadratic form associated to $\mathscr {L}_{\Omega ,B}$ can be written as

$$\begin{aligned} \mathscr {Q}[\Psi ] := \int _\Omega | d_{\textbf{A}}^\times \Psi |^2 {\,\textrm{d}}x, \qquad \textrm{dom}\,\mathscr {Q} := H^1_0(\Omega ). \end{aligned}$$

Now, writing $v = e^{-B \varphi ^\Omega } \Psi$ for $\Psi \in H^1_0(\Omega )$, we have $v\in H^1_0(\Omega )$ and $d_{\textbf{A}}^\times \Psi = - 2 i e^{B \varphi ^\Omega } \partial _{\bar{z}} v$. This is due to our specific choice of gauge. In particular, by the min-max principle we have

3.2 $$\begin{aligned} \lambda _n(\Omega ,B) = \inf _{\begin{array}{c} V \subset H^1_0(\Omega ) \\ \dim V =n \end{array}} \sup _{v \in V\setminus \{0\}} \frac{\displaystyle 4 \int _\Omega | \partial _{\bar{z}} v |^2 e^{2B \varphi ^\Omega } {\,\textrm{d}}x }{\displaystyle \int _\Omega |v|^2 e^{2B \varphi ^\Omega } {\,\textrm{d}}x}. \end{aligned}$$

*Step 2: Construction of quasimodes.* We consider the following space of trial functions, $V= {\textrm{span}} \{v_0, \dots , v_{n-1}\}$ with

$$\begin{aligned} v_k(x) = \chi _B(x) B^{(k+1)/2} (x_1+ix_2)^k e^{- B \varphi ^{\Omega }_\textrm{m}}, \end{aligned}$$

where the cut-off function $\chi _B:\Omega \rightarrow {\mathbb R}$ is defined as

3.3 $$\begin{aligned} \chi _B(x) := \chi \big (B\cdot \textrm{dist}\,(x,\partial \Omega )\big ) \end{aligned}$$

with some $\chi \in C^\infty (\overline{{\mathbb R}}_+)$ satisfying $0\le \chi \le 1$, $\chi (0) = 0$ and $\chi (t) = 1$ for all $t \ge 1$. Here $\textrm{dist}\,(\cdot ,\partial \Omega )$ stands for the usual distance function to the boundary of $\Omega$. This distance function is known to be 1-Lipschitz [19, Section 3]. Hence, we infer that $\chi _B$ is Lipschitz continuous up to the boundary of $\Omega$. In the following, we will use the space *V* in (3.2) to obtain an upper bound on $\lambda _n(\Omega ,B)$. Therefore, we need to get lower bounds on the norm of any $v\in V$ (Step 3), and upper bounds on the energy of $v_k$ (Step 4). As a by-product of Step 3, we will also prove that *V* is indeed *n*-dimensional.

*Step 3: Lower bounds on the norm.* When $v = \sum _{k=0}^{n-1} \alpha _k v_k$ with some complex coefficients $\{\alpha _k\}_{k=0}^{n-1}$, we can estimate its norm,

$$\begin{aligned} \int _\Omega |v|^2 e^{2B \varphi ^\Omega } {\,\textrm{d}}x&= \int _\Omega \chi _B(x)^2 \Big | \sum _{k=0}^{n-1} \alpha _k B^{k/2}(x_1+ix_2)^k \Big |^2 e^{-2B( \varphi ^\Omega _\textrm{m} - \varphi ^\Omega )}B {\,\textrm{d}}x, \\&\ge \int _{\mathcal {D}(0,B^{- \frac{1}{2}})} \Big | \sum _{k=0}^{n-1} \alpha _k B^{k/2}(x_1+ix_2)^k \Big |^2 e^{-\varepsilon B |x|^2}B {\,\textrm{d}}x, \end{aligned}$$

where we restricted the integral to a small disk centred at the origin 0 with the radius $B^{-\frac{1}{2}}$ (provided that *B* is large enough), and used the fact that the Hessian of $\varphi ^\Omega$ at the point of its maximum (the origin) is negative definite; here $\varepsilon$ is some positive constant whose existence is guaranteed, but whose precise admissible value is not essential for what follows. Then making a change of variable $y = \sqrt{B} x$ we get

$$\begin{aligned} \int _\Omega |v|^2 e^{2B \varphi ^\Omega } {\,\textrm{d}}x \ge \int _{\mathcal {D}(0,1)} \Big |\sum _{k=0}^{n-1} \alpha _k (y_1+iy_2)^k \Big |^2 e^{-\varepsilon |y|^2} {\,\textrm{d}}y. \end{aligned}$$

Note that the quantity

$$\begin{aligned} N(P):= \left( \int _{\mathcal {D}(0,1)} | P(y_1 + i y_2) |^2 e^{-\varepsilon |y|^2} {\,\textrm{d}}y \right) ^{\frac{1}{2}} \end{aligned}$$

is a norm on the space of polynomials of degree $\le n-1$. Since this is a finite dimensional space, this norm is equivalent to the Euclidean norm of the coefficients: there is a constant $K_{n}>0$ such that

$$\begin{aligned} \int _{\mathcal {D}(0,1)} \Big |\sum _{k=0}^{n-1} \alpha _k (y_1+iy_2)^k \Big |^2 e^{-\varepsilon |y|^2} {\,\textrm{d}}y \ge K_{n} \sum _{k=0}^{n-1}|\alpha _k|^2, \end{aligned}$$

and $K_{n}$ is independent of the coefficients $\alpha _k$. We deduce that

3.4 $$\begin{aligned} \int _\Omega |v|^2 e^{2B \varphi ^\Omega } {\,\textrm{d}}x \ge K_n \sum _{k=0}^{n-1}|\alpha _k|^2. \end{aligned}$$

In particular, we have shown as a by-product that $\dim V = n$.

*Step 4: Upper bounds on the energy.* We can also estimate the numerator in the min-max characterisation of $\lambda _{n}(\Omega ,B)$ evaluated on the trial function of $v_k$. Since $\partial _{\bar{z}} (x_1+ix_2)^k=0$, we have

$$\begin{aligned} 4 \int _\Omega | \partial _{\bar{z}} v_k |^2 e^{2B \varphi ^\Omega } {\,\textrm{d}}x =B^{k+1}e^{-2 B \varphi _\textrm{m}^\Omega }\int _\Omega | \nabla \chi _B |^2 e^{2B \varphi ^\Omega } {\,\textrm{d}}x . \end{aligned}$$

The gradient of $\chi _B$ is supported on $B^{-1}$-neighbourhood of $\partial \Omega$

$$ \hat{\Omega }_{B^{-1}}:=\{x\in \Omega :\textrm{dist}\,(x,\partial \Omega )\le B^{-1}\},$$

and there exists a constant $C >0$ such that $|\nabla \chi _B| \le CB$ for all sufficiently large $B >0$. Moreover, since $\varphi ^\Omega$ vanishes on the boundary, we have $\varphi ^\Omega \le C' B^{-1}$ on $\hat{\Omega }_{B^{-1}}$ with some constant $C' > 0$ (It follows from boundedness of $\nabla \varphi ^\Omega$, see [46, Eq. 6.12] or [30]). Thus, we can find a constant $L_k >0$ (independent of *B*) such that

3.5 $$\begin{aligned} 4 \int _\Omega | \partial _{\bar{z}} v_k |^2 e^{2B \varphi ^\Omega } {\,\textrm{d}}x \le L_k B^{k+3} e^{-2B \varphi _\textrm{m}^\Omega } | \hat{\Omega }_{B^{-1}}|. \end{aligned}$$

Note that $|\hat{\Omega }_{B^{-1}}| \le C'' B^{-1}$ since any convex domain has Lipschitz boundary. Coming back to (3.5), we obtain

3.6 $$\begin{aligned} 4 \int _\Omega | \partial _{\bar{z}} v_k |^2 e^{2B \varphi ^\Omega } {\,\textrm{d}}x \le C'' L_k B^{k+2} e^{-2B \varphi _{\textrm{m}}^\Omega }. \end{aligned}$$

*Step 5: Conclusion.* When $v = \sum _{k=0}^{n-1} \alpha _k v_k$ is an arbitrary element of $V\setminus \{0\}$, we deduce from (3.6) and the triangle inequality that

$$\begin{aligned} 4 \int _\Omega | \partial _{\bar{z}} v |^2 e^{2B \varphi ^\Omega } {\,\textrm{d}}x \le L_n' B^{n+1} e^{-2B \varphi _\textrm{m}^\Omega } \sum _{k=0}^{n-1} |\alpha _k|^2, \end{aligned}$$

with a new positive constant $L_n'$. Combining with (3.4) we obtain, for all $v \in V\setminus \{0\}$,

$$\begin{aligned} \frac{\displaystyle 4 \int _\Omega | \partial _{\bar{z}} v |^2 e^{2B \varphi ^\Omega } {\,\textrm{d}}x }{\displaystyle \int _\Omega |v|^2 e^{2B \varphi ^\Omega } {\,\textrm{d}}x} \le \frac{L_n'}{K_n} B^{n+1} e^{-2B \varphi _\textrm{m}^\Omega } \end{aligned}$$

and the estimate on $\lambda _n(\Omega ,B)$ follows from the min-max principle (3.2). $\square$

It is also possible to get lower bounds on the magnetic eigenvalues that are uniform with respect to $\Omega$, and that capture the exponential behaviour as $B \rightarrow \infty$.

### Lemma 8

( [28, Theorem 3.1]) Assume that $\Omega \subset {\mathbb R}^2$ is a bounded simply connected domain of unit area. Then, for all $B \ge 0$,

$$\begin{aligned} \lambda _1(\Omega ,B) \ge \lambda _1(\mathbb {D},0) e^{-2B \varphi _\textrm{m}^\Omega }. \end{aligned}$$

We are now in position to prove Theorem 1.

### Proof of Theorem 1

Let $n\in {\mathbb N}$ be fixed. The upper bound from Lemma 7 applied to $\mathbb {D}$ and the uniform lower bound from Lemma 8 applied to $\Omega$ give for all $B \ge M_n(\mathbb {D})$

3.7 $$\begin{aligned} \frac{\lambda _n(\Omega ,B)}{\lambda _n(\mathbb {D},B)} \ge \frac{\lambda _1(\mathbb {D},0) e^{2B (\varphi _\textrm{m} ^\mathbb {D}- \varphi _\textrm{m}^\Omega )}}{C_n(\mathbb {D}) B^{n+1}}. \end{aligned}$$

We then use the quantitative estimate on the torsion function (Lemma 4) to obtain for all $B \ge M_n(\mathbb {D})$

$$\begin{aligned} \frac{\lambda _n(\Omega ,B)}{\lambda _n(\mathbb {D},B)}&\ge \frac{\lambda _1(\mathbb {D},0) e^{2c B \alpha (\Omega )^3}}{C_n(\mathbb {D}) B^{n+1}} = \frac{\lambda _1(\mathbb {D},0)}{C_n(\mathbb {D})} e^{2cB \alpha (\Omega )^3 - (n+1) \ln B} \\&\ge \frac{\lambda _1(\mathbb {D},0)}{C_n(\mathbb {D})}\left( 2 c B \alpha (\Omega )^3 - (n+1) \ln B\right) , \end{aligned}$$

where we used the inequality $e^x \ge x$ in the last step. Note that there exists $B_n > M_n(\mathbb {D})$ and $c_n > 0$ which only depend on the index *n* of the eigenvalue such that

$$ \frac{\lambda _1(\mathbb {D},0)}{C_n(\mathbb {D})}\left( 2 c B \alpha (\Omega )^3 - (n+1) \ln B\right) \ge 1+ c_n B\alpha (\Omega )^3 -\frac{\ln B}{c_n} $$

for all $B \ge B_n$. Thus, the claim of the theorem follows. $\square$

Following the same circle of ideas as in the proof of Theorem 1 we can prove Theorem 2.

### Proof of Theorem 2

Let $n\in {\mathbb N}$ be fixed. The upper bound from Lemma 7 applied to the square ${\textsf {R}}_1$, and the lower bound from Lemma 8 applied to the rectangle ${\textsf {R}}_a$ yield for all $B \ge M_n({\textsf {R}}_1)$

$$\begin{aligned} \frac{\lambda _n(\textsf {R}_a ,B)}{\lambda _n(\textsf {R}_1,B)} \ge \frac{\lambda _1(\mathbb {D},0) e^{2B (\varphi _\textrm{m}^{\textsf {R}_1} - \varphi _\textrm{m}^{\textsf {R}_a})} }{C_n({\textsf {R}}_1) B^{n+1}}. \end{aligned}$$

By Theorem 5 we have

$$\begin{aligned} \varphi _\textrm{m}^{\textsf {R}_1} - \varphi _\textrm{m}^{\textsf {R}_a} \ge \frac{(a^2-1)^2}{24(1+a^4)}. \end{aligned}$$

We deduce that

$$\begin{aligned} \frac{\lambda _n(\textsf {R}_a ,B)}{\lambda _n(\textsf {R}_1,B)} \ge \frac{\lambda _1(\mathbb {D},0)}{C_n({\textsf {R}}_1) } \left( B\frac{(a^2-1)^2}{12(1+a^4)} -(n+1) \ln B\right) , \end{aligned}$$

where we again used the inequality $e^x \ge x$. Clearly, there exist $c_n>0$, $B_n > M_n(\textsf {R}_1)$ such that

$$ \frac{\lambda _1(\mathbb {D},0)}{C_n({\textsf {R}}_1) } \left( B\frac{(a^2-1)^2}{12(1+a^4)} -(n+1) \ln B\right) \ge 1 + c_n B \frac{(a^2-1)^2}{1+a^4} - \frac{\ln B}{c_n} $$

for all $B \ge B_n$ and the claim of the theorem follows. $\square$

## Magnetic Dirac Operator with Infinite Mass Boundary Conditions

In this section, we consider the magnetic Dirac operator $\mathscr {D}_{\Omega ,B}$ with infinite mass boundary conditions on a bounded planar simply-connected $\mathscr {C}^2$-smooth domain $\Omega$. We prove Theorem 3 on the asymptotic minimizers of the positive eigenvalues. Since the domain $\Omega$ is simply-connected, we can choose any gauge $\textbf{A}$ generating the homogeneous magnetic field *B*. It is convenient to use the following gauge, defined through the torsion function,

$$\begin{aligned} A_1 = B \partial _2 \varphi ^\Omega , \qquad A_2 = - B \partial _1 \varphi ^\Omega , \end{aligned}$$

where $\varphi ^\Omega$ is as before the torsion function of $\Omega$. In fact, the positive eigenvalues of $\mathscr {D}_{\Omega ,B}$ can be efficiently characterised by a non-linear variational principle. In order to formulate it, we need to introduce several additional function spaces. Namely, the magnetic Hardy space

$$ \mathscr {H}_\textbf{A}^2(\Omega ) := \big \{u\in L^2(\Omega ):d^\times _\textbf{A} u = 0, \, u|_{\partial \Omega }\in L^2(\partial \Omega )\big \} $$

and the Hilbert space

$$ \mathfrak {H}_\textbf{A}(\Omega ) := H^1(\Omega ) + \mathscr {H}_\textbf{A}^2(\Omega ) $$

with the inner product

$$ \langle u,v\rangle _{\mathfrak {H}_\textbf{A}(\Omega )} := \langle u,v\rangle _{L^2(\Omega )}+ \langle d_\textbf{A}^\times u,d^\times _\textbf{A}v\rangle _{L^2(\Omega )}+ \langle u|_{\partial \Omega },v|_{\partial \Omega }\rangle _{L^2(\partial \Omega )}. $$

In fact, $\mathscr {H}_\textbf{A}^2(\Omega )$ with the scalar product $\langle u|_{\partial \Omega },v|_{\partial \Omega }\rangle _{L^2(\partial \Omega )}$ is a Hilbert space (see [8, Section 2.1] for details and more properties of this space). Then we have the following non-linear min-max principle, for all $n\in {\mathbb N}$,

4.1 $$\begin{aligned} \lambda _n^+(\Omega ,B) = \inf _{{\begin{smallmatrix} \mathcal {L}\subset {\mathfrak H}_{\textbf{A}}(\Omega )\\ \dim \mathcal {L}= n\end{smallmatrix}}}\sup _{u\in \mathcal {L}\setminus \{0\}} \frac{\Vert u\Vert ^2_{L^2(\partial \Omega )} + \sqrt{\Vert u\Vert ^4_{L^2(\partial \Omega )}+ 4\Vert u\Vert ^2_{L^2(\Omega )} \Vert d_\textbf{A}^\times u\Vert ^2_{L^2(\Omega )}}}{2\Vert u\Vert ^2_{L^2(\Omega )}}. \end{aligned}$$

This principle for $\lambda _1^+(\Omega ,0)$ appeared for the first time in [3, Theorem 4]. The magnetic case and the higher eigenvalues are covered by a more general result in [8, Theorem 1.10].

In the analysis of magnetic Dirac eigenvalues we will employ certain geometric bounds on these eigenvalues in terms of the torsion function. The first bound in the case of the disk is a direct consequence of the general result in [8, Theorem 1.11].

### Lemma 9

For any $n\in {\mathbb N}$, there exist constants $C_n, M_n > 0$ such that

$$ \lambda _n^+(\mathbb {D},B) \le C_n B^n e^{-2B \varphi ^{\mathbb {D}}_\textrm{m}} $$

holds for all $B \ge M_n$.

The second estimate is a lower bound for general domains and its proof relies on an adaptation of the trick in [28] (Lemma 8) to the non-linear variational characterisation of the magnetic Dirac eigenvalues.

### Lemma 10

Let $\Omega \subset {\mathbb R}^2$ be a bounded, simply-connected domain with $\mathscr {C}^2$ boundary. Then for any $B > 0$ we have

$$ \lambda _1^+(\Omega ,B) \ge \lambda _1^+(\Omega ,0) e^{-2B\varphi ^\Omega _\textrm{m}}. $$

### Proof

First, we observe that the bounded and boundedly invertible multiplication operator ${\textsf {M}}u:= e^{-B\varphi ^\Omega } u$ acting in $L^2(\Omega )$ is a bijection between ${\mathfrak H}_\textbf{A}(\Omega )$ and ${\mathfrak H}_0(\Omega )$. Indeed, for any $u\in {\mathfrak H}_\textbf{A}(\Omega )$ we have a decomposition $u = u_0 + u_1$, where $u_0\in H^1(\Omega )$ and $u_1\in L^2(\Omega )$ satisfies $d_\textbf{A}^\times u_1 = 0$ and $u_1|_{\partial \Omega }\in L^2(\partial \Omega )$. By regularity of the torsion function we have ${\textsf {M}}u_0 \in H^1(\Omega )$. Moreover, it holds that $u_1|_{\partial \Omega } = {\textsf {M}}u_1|_{\partial \Omega }\in L^2(\partial \Omega )$, where we used that torsion function vanishes on the boundary of the domain $\Omega$. Finally, we also notice that for any $w\in L^2(\Omega )$ with $d_\textbf{A}^\times w\in L^2(\Omega )$ we have

4.2 $$\begin{aligned} d_0^\times {\textsf {M}}w = e^{-B\varphi ^\Omega }\Big (-2i \partial _{\overline{z}} w + i B(\partial _1\varphi ^\Omega ) w - B(\partial _2\varphi ^\Omega ) w\Big ) = e^{-B\varphi ^\Omega } d_\textbf{A}^\times w. \end{aligned}$$

Thus, we conclude that ${\textsf {M}}u ={\textsf {M}}u_0 + {\textsf {M}}u_1\in {{\mathfrak H}_0}(\Omega )$, since ${\textsf {M}}u_0\in H^1(\Omega )$ and by the above identity ${\textsf {M}}u_1\in \mathscr {H}_{0}^2(\Omega )$. Hence, we have established that ${\textsf {M}}$ maps ${\mathfrak H}_{\textbf{A}}(\Omega )$ into ${{\mathfrak H}_0}(\Omega )$. Analogously, one can check that ${\textsf {M}}^{-1}$ maps ${{\mathfrak H}_0}(\Omega )$ into ${\mathfrak H}_{\textbf{A}}(\Omega )$. Thus, we conclude that ${\textsf {M}}$ is a bijection between ${\mathfrak H}_\textbf{A}(\Omega )$ and ${\mathfrak H}_0(\Omega )$. Hence, by the variational characterisation (4.1) we get

$$ \begin{aligned} \lambda _1^+(\Omega ,B)&= \inf _{u\in {\mathfrak H}_{\textbf{A}}(\Omega )\setminus \{0\}} \frac{\Vert u\Vert ^2_{L^2(\partial \Omega )} + \sqrt{\Vert u\Vert ^4_{L^2(\partial \Omega )}+ 4\Vert u\Vert ^2_{L^2(\Omega )} \Vert d_\textbf{A}^\times u\Vert ^2_{L^2(\Omega )}}}{2\Vert u\Vert ^2_{L^2(\Omega )}} \\&=\inf _{v\in {\mathfrak H}_0(\Omega )\setminus \{0\}} \frac{\Vert v\Vert ^2_{L^2(\partial \Omega )} + \sqrt{\Vert v\Vert ^4_{L^2(\partial \Omega )}+ 4\Vert e^{B\varphi ^\Omega }v\Vert ^2_{L^2(\Omega )} \Vert e^{B\varphi ^\Omega }d_0^\times v\Vert ^2_{L^2(\Omega )}}}{2\Vert e^{B\varphi ^\Omega } v\Vert ^2_{L^2(\Omega )}} \\&\ge e^{-2B\varphi ^\Omega _\textrm{m}}\inf _{v\in {\mathfrak H}_0(\Omega )\setminus \{0\}} \frac{\Vert v\Vert ^2_{L^2(\partial \Omega )} + \sqrt{\Vert v\Vert ^4_{L^2(\partial \Omega )}+ 4\Vert v\Vert ^2_{L^2(\Omega )} \Vert d_0^\times v\Vert ^2_{L^2(\Omega )}}}{2\Vert v\Vert ^2_{L^2(\Omega )}} \\&= e^{-2B\varphi ^\Omega _\textrm{m}}\lambda _1^+(\Omega ,0), \end{aligned} $$

where we used the properties of the mapping ${\textsf {M}}$, in particular, we employed the identity (4.2). $\square$

We also recall a lower bound on $\lambda _1^+(\Omega ,0)$ for bounded $\mathscr {C}^2$-smooth simply-connected domains,

4.3 $$\begin{aligned} \lambda _1^+(\Omega ,0) \ge \sqrt{ \frac{2\pi }{|\Omega |}}, \end{aligned}$$

which is proven in [13, Theorem 1], see also [44, Theorem 1].

The proof of Theorem 3 is then identical to the one of Theorem 1, using the upper bound in Lemma 9, the lower bound in Lemma 10, and the quantitative estimate on the torsion function, Lemma 4. For the sake of completeness, we provide it below.

### Proof of Theorem 3

Let $n\in {\mathbb N}$ be fixed. The upper bound from Lemma 9 for the disk and the uniform lower bound from Lemma 10 applied to $\Omega$ and combined with (4.3) give for all $B \ge M_n$

4.4 $$\begin{aligned} \frac{\lambda _n^+(\Omega ,B)}{\lambda _n^+(\mathbb {D},B)} \ge \frac{\sqrt{2\pi } e^{2B (\varphi _\textrm{m} ^\mathbb {D}- \varphi _\textrm{m}^\Omega )}}{ C_n B^n}. \end{aligned}$$

We then use the quantitative estimate on the torsion function (Lemma 4) to obtain for all $B \ge M_n$

$$\begin{aligned} \frac{\lambda _n^+(\Omega ,B)}{\lambda _n^+(\mathbb {D},B)}&\ge \frac{\sqrt{2\pi } e^{2c B \alpha (\Omega )^3}}{ C_n B^n} = \frac{\sqrt{2\pi }}{C_n} e^{2cB \alpha (\Omega )^3 - n\ln B} \ge \frac{\sqrt{2\pi }}{ C_n} \left( 2 c B \alpha (\Omega )^3 - n \ln B\right) , \end{aligned}$$

where we used the inequality $e^x \ge x$ in the last step. Thus, the claim of the theorem follows from the above inequality, since there exist constants $c_n>0$, $B_n> M_n$ such that

$$ \frac{\sqrt{2\pi }}{C_n} \left( 2 c B \alpha (\Omega )^3 - n \ln B\right) \ge 1 + c_n B \alpha (\Omega )^3 - \frac{\ln B}{c_n} $$

for all $$B \ge B_n$$. $$\square $$

## Data Availability

There is no data related to this work.

## Proof of Lemma 4

For $t \ge 0$, define the level sets $U_t = \lbrace x \in \Omega :\varphi ^\Omega (x) \ge t \rbrace$, and $\mu (t) = | U_t |$. We start by bounding the perimeter $P(U_t)$ in terms of $\mu (t)$. By Sard’s Theorem, for almost all *t* the boundary $\partial U_t = \lbrace \varphi ^\Omega = t \rbrace$ is smooth, and therefore

$$\begin{aligned} P(U_t)^2 = | \lbrace \varphi ^\Omega = t \rbrace |^2 = \left( \int _{\lbrace \varphi ^\Omega = t \rbrace } {\,\textrm{d}}\mathcal {H}^1 \right) ^2, \end{aligned}$$

where ${\,\textrm{d}}\mathcal {H}^1$ is the measure induced by the Lebesgue measure^3^ on the curve $\partial U_t$. By Sard’s Theorem again, $\nabla \varphi ^\Omega$ is non-vanishing on $\lbrace \varphi ^\Omega = t \rbrace$ for almost all *t*. Therefore we can use the Cauchy-Schwarz inequality to get

A.1 $$\begin{aligned} P(U_t)^2 \le \left( \int _{\lbrace \varphi ^\Omega = t \rbrace } | \nabla \varphi ^\Omega |{\,\textrm{d}}\mathcal {H}^1\right) \left( \int _{\lbrace \varphi ^\Omega = t \rbrace } \frac{1}{|\nabla \varphi ^\Omega |}{\,\textrm{d}}\mathcal {H}^1\right) , \end{aligned}$$

for almost all $t\ge 0$. The co-area formula gives

$$\begin{aligned} \mu (t) = \int _{U_t} {\,\textrm{d}}x = \int _t^{\varphi _\textrm{m}^\Omega } \int _{\lbrace \varphi ^\Omega = s \rbrace } \frac{1}{|\nabla \varphi ^\Omega |}{\,\textrm{d}}\mathcal {H}^1 {\,\textrm{d}}s \end{aligned}$$

and therefore

A.2 $$\begin{aligned} \mu '(t) = -\int _{\lbrace \varphi ^\Omega = t \rbrace } \frac{1}{|\nabla \varphi ^\Omega |}{\,\textrm{d}}\mathcal {H}^1, \end{aligned}$$

for almost all $t\ge 0$. Moreover, by definition of $\varphi ^\Omega$ we have

$$\begin{aligned} \mu (t) = \int _{\lbrace \varphi ^\Omega \ge t \rbrace } - \Delta \varphi ^\Omega {\,\textrm{d}}x, \end{aligned}$$

and since the inward-pointing normal to $\partial U_t$ is $\nabla \varphi ^\Omega / | \nabla \varphi ^\Omega |$, we can use Stokes Theorem to get

A.3 $$\begin{aligned} \mu (t) = \int _{\lbrace \varphi ^\Omega = t \rbrace } \nabla \varphi ^\Omega \cdot \frac{\nabla \varphi ^\Omega }{|\nabla \varphi ^\Omega |} {\,\textrm{d}}\mathcal {H}^1 = \int _{\lbrace \varphi ^\Omega = t \rbrace } |\nabla \varphi ^\Omega | {\,\textrm{d}}\mathcal {H}^1, \end{aligned}$$

for almost all $t\ge 0$. Then, we use (A.2) and (A.3) to rewrite (A.1) as

A.4 $$\begin{aligned} P(U_t)^2 \le - \mu (t) \mu '(t), \end{aligned}$$

for almost all $t\ge 0$. We now use the quantitative isoperimetric inequality [25, Theorem 1.1] for $U_t$, which gives a universal constant $\textsf {c}_\textrm{iso} >0$ such that

$$\begin{aligned} P(U_t)^2 \ge 4\pi |U_t| \big ( 1 + \textsf {c}_\textrm{iso} \alpha (U_t)^2 \big ), \end{aligned}$$

and we deduce that

$$\begin{aligned} 4 \pi ( 1+ \textsf {c}_\textrm{iso} \alpha (U_t)^2 ) \le - \mu '(t), \end{aligned}$$

for almost all $t \in (0, \varphi ^\Omega _\textrm{m})$. Integrating over the whole interval we find

$$\begin{aligned} 4 \pi \varphi ^\Omega _\textrm{m} + 4 \pi \textsf {c}_\textrm{iso} \int _0^{\varphi ^\Omega _\textrm{m}} \alpha (U_t)^2 {\,\textrm{d}}t \le \mu (0) = |\Omega |. \end{aligned}$$

Note that the torsion function of the disk $\mathcal {D}$ is the radial function $r \mapsto \frac{|\Omega |}{4\pi } - \frac{r^2}{4}$. Therefore,

A.5 $$\begin{aligned} \varphi ^{\mathcal {D}}_\textrm{m} - \varphi ^{\Omega }_\textrm{m} \ge \textsf {c}_\textrm{iso} \int _0^{\varphi _\textrm{m}^\Omega } \alpha (U_t)^2 {\,\textrm{d}}t. \end{aligned}$$

Now consider the threshold $s_\Omega$ from which $\mu (t)$ gets far from $|\Omega |$, namely

A.6 $$\begin{aligned} s_\Omega = \sup \mathscr {A}, \qquad \mathscr {A} = \Big \lbrace t \ge 0:\mu (t) \ge |\Omega | \Big ( 1 - \frac{\alpha (\Omega )}{4} \Big ) \Big \rbrace . \end{aligned}$$

For $t \in \mathscr {A}$ we have $\frac{| \Omega \setminus U_t |}{|\Omega |} \le \frac{1}{4} \alpha (\Omega )$, which implies $\alpha (U_t) \ge \frac{\alpha (\Omega )}{2}$ by standard properties of the Fraenkel asymmetry [9, Lemma 2.8]. Thus, equation (A.5) gives

A.7 $$\begin{aligned} \varphi ^{\mathcal {D}}_\textrm{m} - \varphi ^{\Omega }_\textrm{m} \ge \frac{\textsf {c}_\textrm{iso}}{4} s_\Omega \alpha (\Omega )^2. \end{aligned}$$

We now consider two complementary cases.

(i) If $\alpha (\Omega ) \le \frac{32 \pi }{|\Omega |} ( \varphi ^{\mathcal {D}}_\textrm{m} - \varphi ^{\Omega }_\textrm{m} )$, then Lemma 4 is obvious since $\alpha (\Omega ) \ge \frac{1}{4}\alpha (\Omega )^3$.
(ii) If $\alpha (\Omega ) \ge \frac{32 \pi }{|\Omega |} ( \varphi ^{\mathcal {D}}_\textrm{m} - \varphi ^{\Omega }_\textrm{m} )$, we use that $\varphi ^\Omega - s_\Omega$ is the torsion function on $U_{s_\Omega }$. Therefore, its maximum is smaller than the maximum of the torsion of the disk of volume $|U_{s_\Omega }|$:
  $$\begin{aligned} \varphi _\textrm{m}^\Omega - s_\Omega \le \frac{|U_{s_\Omega }|}{4\pi } = \frac{|\Omega |}{4\pi } \Big ( 1 - \frac{\alpha (\Omega )}{4} \Big ). \end{aligned}$$
  Since $\varphi _\textrm{m}^{\mathcal {D}} = \frac{|\Omega |}{4\pi }$, we deduce
  $$\begin{aligned} s_\Omega \ge \frac{|\Omega | \alpha (\Omega )}{16\pi } - \big ( \varphi ^{\mathcal {D}}_\textrm{m} - \varphi ^\Omega _\textrm{m} \big ) \ge \frac{|\Omega | \alpha (\Omega )}{32\pi }. \end{aligned}$$
  Then (A.7) gives $\varphi _\textrm{m}^{\mathcal {D}} - \varphi _\textrm{m}^\Omega \ge \frac{\textsf {c}_\textrm{iso}}{128 \pi } |\Omega | \alpha (\Omega )^3$, which concludes the proof.

Finally, we remark that thanks to the bound $\textsf {c}_\textrm{iso} \ge \frac{1}{8}$ given in [40, Theorem 1] we can state that inequality in an explicit form,

$$ \varphi _\textrm{m}^{\mathcal {D}} - \varphi _\textrm{m}^\Omega \ge \frac{1}{1024 \pi } |\Omega | \alpha (\Omega )^3. $$

## Footnotes

1 This operator is also one of the components of the magnetic Dirichlet-Pauli operator (see [7]).

2 In fact, there are some assumptions on $\Omega$ in [7], including smoothness, and uniqueness of the maximum of $\varphi ^\Omega$. This last property is always satisfied for convex domains (see [36, Theorem 1]).
